# Detrimental role of humoral signalling in cardio-renal cross-talk

**DOI:** 10.1186/cc13704

**Published:** 2014-01-22

**Authors:** Vincenzo Cantaluppi, Sergio Dellepiane, Alessandro D Quercia, Silvia Ferrario

**Affiliations:** 1 Nephrology, Dialysis and Kidney Transplantation Unit, Department of Medical Sciences, University of Torino, Azienda Ospedaliera Città della Salute e della Scienza ‘Molinette’, Torino, 10126, Italy

## Abstract

In critically ill patients, any acute organ injury is associated with a sudden change of circulating factors that may play a role in distant organ dysfunction through a complex cross-talk. In this issue, Virzì and colleagues discuss the relevance of humoral signalling between heart and kidney, focusing on type 1 and type 3 cardio-renal syndrome. We herein review the mechanisms of heart-kidney cross-talk, discussing the role of circulating detrimental mediators in the pathogenetic mechanisms of cardio-renal syndrome.

## 

The ‘humoral theory’ proposed by the greek physician Hippocrates sustained that the body is composed of the four basic humors, black bile, yellow bile, phlegm and blood. According to this theory, an excess or a deficit of one of these humors leads to a disease state. Nowadays, the role of an imbalance of humoral mediators in different diseases is still present, particularly in the field of multiple organ failure in critical illness. In this issue, Virzì and colleagues demonstrate the relevance of humoral signalling in type 1 and type 3 cardio-renal syndrome (CRS) [1].

Kidney seems to play a key role in the interplay between distant organs: acute kidney injury (AKI) usually develops as a result of the presence of hypoperfusion or a systemic inflammatory reaction caused by a primary injury in the lung, brain, liver or heart [2]. AKI contributes to the development of this deleterious cross-talk through deregulation of the immune system. This effect may be ascribed to the loss of function of tubular cells that are immunologically active operating as antigen-presenting cells and are known to orchestrate the clearance of inflammatory mediators [3,4].

Distant organs continuously communicate through a complex network of extracellular molecules. CRS is defined as a primary disorder of heart or kidney whereby acute or chronic dysfunction originating from one organ may induce acute or chronic dysfunction of another [5]. Type 1 CRS reflects an abrupt worsening of cardiac function that consequently leads to AKI. Type 2 CRS includes chronic abnormalities of cardiac function able to induce progressive chronic kidney disease. Heart and kidney are both supplied by sympathetic and parasympathetic innervations that regulate blood pressure, vascular tone, diuresis, natriuresis and tissue oxygenation. In type 1 and type 2 CRS, the renin-angiotensin-aldosterone system (RAAS) plays a pivotal role in the modulation of renal perfusion pressure and RAAS activation is associated with vasoconstriction mediated by enhanced release of endothelin [6]. In type 2 CRS, RAAS activation induces oxidative stress and release of free oxygen radicals, thus favouring apoptosis and fibrosis with progression of both renal and cardiac dysfunction [7].

Type 3 CRS consists of an acute cardiac dysfunction following AKI: the pathogenetic mechanisms of cardiomyocyte injury after ischemic AKI can be ascribed to apoptosis associated with increased plasma levels of TNF-alpha. Indeed, the selective blockade of TNF-alpha limited cardiac apoptosis [8]. To further support the relevance of humoral signalling in type 3 CRS, Naito and colleagues [9] elegantly demonstrated that AKI sensitizes the kidney to endotoxin-driven production of cytokines and chemokines. This hyper-responsiveness to endotoxin is likely mediated by an increase of histone methylation and consequent recruitment of RNA polymerase II to the TNF-alpha and MCP-1 genes.

In type 4 CRS, the accumulation of water soluble and protein-bound uremic toxins contributes to the typical endothelial dysfunction and vascular calcification of chronic kidney disease patients [10]. The endogenous inhibitor of nitric oxide synthase ADMA, p-cresyl-sulphate and indoxyl-sulphate induce oxidative stress, and endothelial and cardiomyocyte apoptosis [10,11]. Elevated plasma levels of these uremic toxins are associated with increased cardiovascular risk and mortality [12].

Type 5 CRS reflects a systemic condition causing simultaneous cardiac and renal dysfunction. Sepsis, the systemic response to infection, is the main cause of type 5 CRS. The mechanisms of cardiac and renal dysfunction during sepsis are related to the detrimental role of circulating mediators such as bacterial compounds (lipopolysaccharide and inflammatory cytokines (TNF-alpha, interleukin-6)) able to induce apoptotic tissue damage [13].

In CRS, other metabolites, nucleic acids and lipids can be released by different types of activated cells and circulate into the bloodstream free or bound to specific carriers such as extracellular vesicles (EVs). EVs are membrane-delimited vesicles released from the plasma membrane of different cell types and able to transfer proteins, bioactive lipids and genetic information to a target cell [14]. Platelet-derived EVs isolated from plasma of septic patients induce myocardial and endothelial dysfunction through activation of caspase-3 and generation of superoxide, nitric oxide and peroxynitrite [15].

In conclusion, humoral signalling plays a key role in the pathogenesis of heart and kidney injury in CRS. The blockade of this detrimental humoral cross-talk may lead to an improvement of organ failure. This could be obtained by using early biomarkers of disease or by developing new therapeutic approaches aimed to limit the inflammatory response, including blood purification techniques and stem cell-based treatments.

### Abbreviations

AKI: Acute kidney injury; CRS: Cardio-renal syndrome; EV: Extracellular vesicle; RAAS: Renin-angiotensin-aldosterone system; TNF: Tumour necrosis factor.

### Competing interests

The authors declare that they have no competing interests.
